# TcNPR3 from *Theobroma cacao* functions as a repressor of the pathogen defense response

**DOI:** 10.1186/1471-2229-13-204

**Published:** 2013-12-06

**Authors:** Zi Shi, Yufan Zhang, Siela N Maximova, Mark J Guiltinan

**Affiliations:** 1 The Huck Institutes of the Life Sciences, The Pennsylvania State University, University Park, PA 16802, USA; 2 The Department of Plant Science, The Pennsylvania State University, University Park, PA 16802, USA; 3 422 Life Sciences Building, University Park, PA 16802, USA

**Keywords:** Plant defense, *NPR3*, Negative regulator, *Cacao*, *NPR1*

## Abstract

**Background:**

*Arabidopsis thaliana* (Arabidopsis) NON-EXPRESSOR OF PR1 (NPR1) is a transcription coactivator that plays a central role in regulating the transcriptional response to plant pathogens. Developing flowers of homozygous *npr3* mutants are dramatically more resistant to infection by the pathogenic bacterium *Pseudomonas syringae*, suggesting a role of NPR3 as a repressor of NPR1-mediated defense response with a novel role in flower development.

**Results:**

We report here the characterization of a putative *NPR3* gene from the tropical tree species *Theobroma cacao (TcNPR3*). Like in Arabidopsis*, TcNPR3* was constitutively expressed across a wide range of tissue types and developmental stages but with some differences in relative levels compared to Arabidopsis. To test the function of *TcNPR3*, we performed transgenic complementation analysis by introducing a constitutively expressing putative *TcNPR3* transgene into an Arabidopsis *npr3* mutant. *TcNPR3* expressing Arabidopsis plants were partially restored to the WT pathogen phenotype (immature flowers susceptible to bacterial infection). To test *TcNPR3* function directly in cacao tissues, a synthetic microRNA targeting *TcNPR3* mRNA was transiently expressed in cacao leaves using an *Agrobacterium*-infiltration method. *TcNPR3* knock down leaf tissues were dramatically more resistance to infection with *Phytophthora capsici* in a leaf bioassay, showing smaller lesion sizes and reduced pathogen replication.

**Conclusions:**

We conclude that *TcNPR3* functions similar to the Arabidopsis *NPR3* gene in the regulation of the cacao defense response. Since TcNPR3 did not show a perfect complementation of the Arabidopsis NPR3 mutation, the possibility remains that other functions of TcNPR3 remain to be found. This novel knowledge can contribute to the breeding of resistant cacao varieties against pathogens through molecular markers based approaches or biotechnological strategies.

## Background

To effectively combat a wide range of pathogens, plants are able to induce a complex network of defense responses against invasions [1]. Upon activation of the defense response, a large shift in resource allocation results in reduced growth rate and seed production [2,3]. Therefore, defense responses are highly regulated through a complicated network of positive and negative regulatory mechanisms that allow plants to react appropriately to pathogens only when needed [4-6].

The most well-studied regulator of systemic acquired pathogen resistance (SAR) is Non Expressor of PR1 (NPR1), a transcription coactivator that regulates *PR* gene expression in the SA-dependent signaling pathway [7,8]. *Arabidopsis thaliana (*Arabidopsis) *npr1* mutants exhibit higher susceptibility to pathogen infection and impaired expression of *PR* genes [9]. Additional positive regulators have been identified in SA-dependent systemic signaling, including Enhanced Disease susceptibility 1 (EDS1) [10], SA Induction Deficient 1 (SID1) [11] and TGA transcription factors [12].

Several negative regulators of the plant defense response have also been discovered in Arabidopsis [13-18]. The NIMIN proteins show no obvious homology to known transcription factors other than a short EAR motif, a motif found on repressors of ethylene and auxin induced transcription. NIMIN is thought to act as a modulator of PR-1 transcription levels, suppressing its expression during SA stimulated induction. The cpr1 (constitutive expresser of PR1) mutation causes constitutively high SA levels, increased PR1 expression in the absence of pathogen, and thus enhanced disease resistance [16]. SNI1 (suppressor of npr1-1 inducible) was identified as a repressor of genes targeted by NPR1 induction, thus dampening the basal expression levels of downstream PR genes [13,19]. SNI1 is a nuclear protein and it may function via chromatin remodeling through histone modification. Moreover, the WRKY transcription factors WRKY11 and WRKY17 are also involved in negative regulation of defense responses [15]. NPR1 was recently shown to bind to SA and this interaction results in a conformational change which may contribute to the ability of NPR1 to activate transcription [20].

The functions of the Arabidopsis NPR1 paralogs, NPR2, NPR3 and NPR4, are partially known. Various lines of evidence demonstrate that they all act as components of the SA signal transduction pathway [21-24]. NPR3 and NPR4 have both been shown to bind to SA and to function in the regulation of the rate of proteosomal degradation of NPR1 through a Cullin 3/ubiquitinE3 ligase mediated degradation pathway, thus linking SA concentration to the NPR1-SA-dependant transcriptional defense response [24]. NPR3 acts as a repressor of NPR1 function by enhancing NPR1 degradation, while NPR4 acts at lower SA concentrations to enhance NPR1 stability and increasing defense pathway activation. It is possible that all three proteins act as SA receptors working in different mechanisms to co-regulate the transcriptional defense response.

Understanding the mechanisms regulating the defense response has profound implications for agriculture. Such knowledge can be used to design breeding strategies, or to create transgenic plants with enhanced disease resistance against the plethora of plant pathogens. One such disease, witches’ broom disease of *Theobroma cacao (*cacao), is caused by the fungal pathogen *Moniliophthora perniciosa*, is a serious disease in Latin America. Indigenous to the Amazon rainforest, it now occurs in most cocoa growing regions in South America and has became a serious threat to world cocoa production [25,26]. Terminal and axillary buds, flower cushions and developing pods are highly susceptible to witches’ broom infection, resulting in severe yield loss [27]. The genome of this pathogen was recently sequenced [28] and a deeper understanding of the mechanisms of pathogenicity is beginning to emerge [29-32]. In an effort to advance breeding of disease resistant varieties of cacao, we conducted research to further our understanding of the defense response in this tropical tree species.

Several homologs of Arabidopsis defensive proteins were previously identified in cacao after SA/MeJA treatment [33,34] suggesting that a translational biology approach could be used to accelerate our knowledge of the cacao defense pathways. We have previously described the isolation and functional analysis of the cacao *TcNPR1* gene [35] and demonstrated it is a functional ortholog of Arabidopsis NPR1 by restoring the *npr1-2* mutant phenotype in transgenic Arabidopsis. In addition, a phylogenetic analysis of all four of the NPR1-like gene family members in cacao was presented in the supplementary material of Agrout et al. [36], in which the full genome sequence of *Theobroma cacao* was described.

In this manuscript, we present the isolation and functional analysis of the *Theobroma cacao* gene Tc06t011480 (*TcNPR3*) that is homologous to the Arabidopsis NPR3 gene At1G5G45110. The *TcNPR3* gene was capable of partial restoration of the Arabidopsis *npr3-3* mutant disease resistant flower phenotype. Moreover, micro-RNA-mediated knock down of *TcNPR3* expression in cacao leaves was strongly correlated to decreased susceptibility to infection by the cacao pathogen *Phytophthora capsici*. Based on our results, we conclude that *TcNPR3* shares at least some of the central features of Arabidopsis *NPR3,* and plays a role as a negative regulator of the defense response.

## Material and methods

### Genomic resources utilized

Cacao transcriptomes: The Esttik database (http://esttik.cirad.fr/) houses EST discovery data from fifty-six different cDNA libraries constructed from different organs, genotypes and in varying environmental conditions [37]. 454 transcriptome: A cDNA library from six mixed tissues of Criollo genotype (B97-61/B2) was sequenced by Roche/454 technology as previously described [36]. Cacao genome sequence: The genome of *Theobroma cacao* was sequenced and analyzed from a Belizean Criollo genotype (B97-61/B2) and can be accessed via the Gbrowse website (http://cocoagendb.cirad.fr) [36].

### Full length cDNA cloning and genomic DNA cloning of *TcNPR3*

Unless otherwise indicated, all chemicals and supplies were obtained from Sigma-Aldrich, St. Louis, MO. A partial sequence of the *TcNPR3* gene was identified by screening BAC filter arrays constructed using genomic DNA of genotype LCT-EEN 37 obtained from the Clemson University Genomic Institute (http://www.genome.clemson.edu/) using a partial *TcNPR1* sequence as a radio-labeled hybridization probe. Based on its partial sequence, primers were designed to clone the full-length cDNA of *TcNPR3* from genotype Scavina6 (SCA6). Total RNA was isolated from cacao SCA6 stage C leaves as previously described in [33]. Cacao cDNA was synthesized in a final volume of 25 μl from 2 μg of total cacao RNA using M-MLV reverse transcriptase (New England Biolabs, Inc., Ipswich, MA) as described previously [35]. A full-length *TcNPR3* cDNA (starting at the ATG codon and ending at the stop codon of the predicted CDS) was cloned by PCR using 1 μl of ½ diluted cDNA as template and 5 μM of primers were used to include *Kpn*I and *Not*I restriction sites at the 5′- and 3′-ends respectively (*TcNPR3*-5′-*Kpn*I, GCGGTACCATGGCGTATTTATCTGAG; *TcNPR3*-3′-*Not*I, GCGCGGCCGCTCACAATTTTCTGAGC). All synthetic oligonucleotides were purchased from Integrated DNA Technologies, Coralville, ID. PCR was performed using the following conditions: 94°C for 2 min., 32 cycles of 94°C for 30 sec., 60°C for 30 sec., followed by a 5 min. final extension at 72°C. PCR product was resolved on 1% agarose gels, purified with the GENECLEAN II Kit (Q-Biogene Inc., Solon OH) and cloned into the pGEM T-Easy vector (Promega Corporation, Madison WI). Forward and reverse sequencing was performed at the Penn State Genomics Core Facility to verify the sequence. The resulting clone was designated as pGEM-*TcNPR3*. The full-length *TcNPR3* cDNA sequence was used to identify the *TcNPR3* genomic locus in the genome assembly (Tc06t011480) using blastn [38].

### Gene and protein structure and analysis

The *TcNPR3* gene structure was annotated by alignment of the full-length cDNA with the genomic sequence using SPIDEY (http://www.ncbi.nlm.nih.gov/spidey/). Arabidopsis NPR3 protein sequence was retrieved from TAIR (At5g45110) and compared to the conceptual translation of the *TcNPR3* sequence using MUSCLE [39]. Putative functional motifs were identified using Simple Modular Architecture Research Tool (SMART) (http://smart.embl-heidelberg.de/).

### Gene expression measurements

RT-Q-PCR was performed to measure the relative expression of TcNPR3 in different cacao tissues. Basically, total RNA was extracted from leaf stages A, C and E, open flowers, un-open flowers, roots, pod seeds and pod exocarps. Three biological replicates were collected for each tissue. Cacao cDNA was synthesized in a final volume of 25 μl from 2 μg of total cacao RNA using M-MLV reverse transcriptase (New England Biolabs, Inc., Ipswich, MA) following the protocol given in [35].

The primers to detect TcNPR3 transcripts were designed based on the coding sequence of TcNPR3 (TcNPR3-Realtime-5′: GCCAGAGGTTGACAAGACCAAAGG; TcNPR3-Realtime-3′: GTCTCATGTGTAGATCATCAGCCAACG). The 10 μl quantitative RT-Q-PCR mixture contains 4 μl diluted-cDNA (1:50), 5 μl SYBR Green PCR Master Mix (Takara), 0.2 μl Rox, and 0.4 μl each 5 μM primers. Each reaction was performed in duplicates in Roche Applied Biosystem StepOne Plus Realtime PCR System under the following program: 15 min at 94°C, 40 cycle of 15 sec. at 94°C, 20 sec. at 60°C, and 40 sec. at 72°C. The specificity of the primer pairs were examined by RT-Q-PCR visualized on the 2% Agarose Gel and dissociation curve. Based on consistent expression level in the different samples, the cacao tubulin1 (TUB1, Tc06g000360) gene was used as a control and used to normalize expression data (TUB1-5′: GGAGGAGTCTCTATAAGCTTGCAGTTGG and TUB1-3′: ACATAAGCATAGCCAGCTAGAGCCAG).

### Transgenic Arabidopsis genetic complementation

A T-DNA binary vector designed for overexpression of the *TcNPR3* coding sequence (p35S: *TcNPR3*) was created as follows. The *TcNPR3* coding sequence was excised from pGEM-*TcNPR3* vector and cloned into *Kpn*I and *Not*I sites of an intermediate cloning vector (pE2113) between the very strong E12-Ω promoter [40] and a 35S-CaMV terminator. A 3 kb restriction fragment containing this *TcNPR3* gene cassette was excised from pE2113 using *EcoR*I and *Pvu*II and ligated into the *EcoR*I and *Sma*I sites of pCAMBIA-1300 [41]. Ligations were performed for 1 hour at room temperature with 3 units of T4 DNA ligase (Promega Corporation, Madison WI) resulting in p35S:*TcNPR3*. The binary vector p35S:*TcNPR3* containing a plant selectable hygromycin resistance gene was introduced into *Agrobacterium tumefaciens* strain AGL1 by electroporation as previously described [42]. The floral dip method was used to transform Arabidopsis *npr3-3* mutants with p35S:*TcNPR3 *[43]. Seeds were collected for 5 individual transgenic lines and the screening of positive transformants was conducted as in [35]. Flower tissues from six-week old soil-grown Col-0, *npr3-3* mutant and five individual transgenic lines were collected. RNA was extracted from each genotype using RNeasy plant mini kit (QIAGEN, Valencia CA). cDNA was synthesized using M-MLV reverse transcriptase (New England Biolabs, Inc., Ipswich, MA) as in [35]. RT-PCR was performed to identify the heterologous *TcNPR3* expression. *AtUbiquitin* served as a cDNA loading and normalization control. Following primers and conditions were employed: *TcNPR3*-RT5′: TGCTTGTCGACCCGCCATCAATTT; *TcNPR3*-RT3′: AGGTTGTCTCAGCATGTGCTATGTCC (27 cycles of 94 C for 30 sec., 56°C for 30 sec., 72°C for 1 min). Ubiquitin-5′: ACCGGCAAGACCATCACTCT; Ubiquitin-3′: AGGCCTCAACTGGTTGCTGT (22 cycles of 94°C for 30 sec., 54°C for 30 sec., 72°C for 1 min). The PCR products were resolved on 1% agarose gels.

### *Pseudomonas syringae* infection assay

Plants were grown in a Conviron growth chamber at 22°C, with 16 h light/8 h dark cycle 60% humidity and 200 μM/m^2^light intensity (Octron 4100 K Ecologic bulbs). *Pseudomonas syringae* pv. tomato DC3000 cultures were grown on pseudomonas agar with kanamycin (25 ng/μl) and rifampicin (100 ng/μl) at 28°C for two days. The pathogen infection and bacterial bioassay was conducted as previously described in [23].

### Cacao microRNA construct

A 21 nucleotide microRNA of TcNPR3 was designed using the web-based designer at WMD3 (http://wmd3.weigelworld.org/cgi-bin/webapp.cgi?page%20=%20Designer;project%20=%20stdwmd) by choosing cacao EST TcaGI-3.0 database. The miRNA targets 373 bp upstream of the stop codon. The primer sequences were: (capital letters indicates the target sequence of TcNPR3).

I miR-s gaTTTGAACCTTTTGATGCACAAtctctcttttgtattcc

II miR-a gaTTGTGCATCAAAAGGTTCAAAtcaaagagaatcaatga

III miR*s gaTTATGCATCAAAACGTTCAATtcacaggtcgtgatatg

IV miR*a gaATTGAACGTTTTGATGCATAAtctacatatatattcct

A sequence containing an inverted repeat/hairpin loop structure was amplified from pRS300 following the protocol on WMD3 (Stephan Ossowski, Joffrey Fitz, Rebecca Schwab, Markus Riester and Detlef Weigel, personal communication), also as described in [44,45], with the exception of using the following primers: F-SpeI: TACTAGTGGTACCGGGCCCCCC and R-HpaI: GCGTTAACCTAGTGGATCCCCCCATGG instead of oligo A and B, respectively which were used to amplify the completed fragment for cloning into the SpeI/HapI sites of transformation vector pGH00.0126 [46] driven by the 35S promoter.

### Transient gene knockout and detached leaf pathogen bioassay

Vectors pGH00.0126 (control) and pGS12.0225 (including gene cassette 35S: *TcNPR3* microRNA) were transformed in cacao leaves by *Agrobacterium* (AGLI strain) vacuum infiltration. *Agrobacterium* was grown in 523 medium overnight to OD_600_ = 1.0 and induced as described in Maximova et al., [47] for 5 hours. Leaves, at developmental stage C [35], were collected from greenhouse-grown plants, genotype SCA6 and cut perpendicular to the midvein into 2 pieces per leaf. The 2 pieces from each leaf where infected with the control and the treatment vectors individually, for total of 8 leaf pieces per vector. Prior to infection the cut surfaces of the mid-veins and secondary veins were sealed with melted paraffin (Paraplast Plus, McCormick Scientific). The sealed leaf pieces were submerged, abaxial side down, into 30 ml bacteria solution with 0.02% Silwet L-77 (Vac-In-Stuff, added to the bacterial solution after induction) dispensed in Petri dishes (100 mm × 15 mm, VWR International, Radnor PA). The Petri dishes were placed in a 233 mm polycarbonate vacuum desiccator (Bel-Art, Wayne NJ) and vacuum pressure (22 psi) was applied for 2 min followed by a slow release. Leaves were then blotted dry on a paper towels and placed abaxial side up into a Petri dish (100 × 15 mm) containing 6 layers of absorbent paper towels and one Whatman #3 filter paper pre-moistened with 10 ml sterile water. The Petri dishes were sealed with parafilm and incubated at 25°C for 2 days with light intensity of 145 m^-2^ sec.^-1^and 14 h daylight. After two days, two replicate leaf pieces per vector were quickly frozen on liquid nitrogen. Total RNA was extracted as previously described [33] and cDNA was obtained as described above. The relative expression of *TcNPR3* to *TcActin* was measured by RT-Q-PCR using Takara SYBR premix EX TaqII kit (Clontech) according to the user manual with 1:200 dilution of the cDNA template. For each measurement, two biological replicates, each with two technical replicates were performed for both genes using the primers described above.

Cacao pathogen *Phytophthora capsici* was activated on Petri dishes containing 10% V8 medium (100 ml/l V8 juice, 3 g/l calcium carbonate and 15 g/l bacto agar) for two days at 27°C, 12 h daylight. Attached leaf pathogen bioassay was performed as previously described [23] on the remaining six leaf pieces. The right half of each leaf portion was inoculated with 3 agar plugs containing actively growing *Phytophthora capsici* mycelium and the left half was inoculated with sterile agar plugs as negative control. Inoculated leaves were incubated at 27°C and 12 h day/light cycle for three days before the evaluation of disease symptoms. Photographic images of the leaves were taken with a Nikon D90 camera and average lesion sizes were determined using ImageJ software tools (Imagej.nih.gov). Average lesion sizes are calculated from 18 measurements (6 leaf pieces, 3 replicates) and significance was determined by single factor ANOVA. To measure pathogen replication as a proxy of virulence, the ratio of *Phytophthora* DNA to cacao DNA was measured by RT-Q-PCR as follows. Tissue samples including the lesions (1.4 cm^2^ surrounding the inoculation site) were excised from the infected leaves and used for genomic DNA extraction using a Tissue Lyzer homogenizer and the DNeasy plant mini kit (Qiagen). The relative amount of *Phytophthora capsici* genomic DNA in leaf disks was measured by amplification of a *PcActin* gene (primer set F: GACAACGGCTCCGGTATGTGCAAGG and R: GTCAGCACACCACGCTTGGACTG) and *TcActin7* (Tc01t010900) (primer set F: AGCTGAGAGATTCCGTTGTCCAGA and R: CCCACATCAACCAGACTTTGAGTTC) were used as pathogen and host targets respectively. DNA RT-Q-PCR was preformed as described in Wang et al. [48], using a ABI 7300 Real-Time PCR System (Penn State Genomics Core Facility), and the ratio of *P. capsici* DNA to cacao DNA in the infected tissues was calculated as two to the power of the difference between Ct numbers (2^(Ct-P. capsici - Ct-T.cacao)^).

### Accession numbers

Sequence data from this article can be found in the Arabidopsis Genome Initiative, GenBank databases or cacao genome browser (http://cocoagendb.cirad.fr/gbrowse/cgi-bin/gbrowse/theobroma/) under the following accession numbers: At5g45110 (*NPR3*), At3g52590 (*ubiquitin*), BT031870.1(*Phytophthora Actin*), Tc06t011480/ JX983187 (*TcNPR3*), Tc01t010900 (*TcActin*) and Tc06g000360 (*TcTUB1*).

## Results

### Isolation of a putative *TcNPR3* gene

Initially, a partial *TcNPR3* gene was identified by screening a BAC library by hybridization with a partial *TcNPR1* sequence. Based on this sequence, PCR primers were used to amplify cDNA isolated from cacao genotype Scavina6 (SCA6) stage C leaves. A fragment of 1764 bp was isolated, cloned into pGEM vector and sequenced to reveal an intact coding sequence of the expected length and with high homology to the Arabidopsis *NPR3* gene.

**Figure 1:**
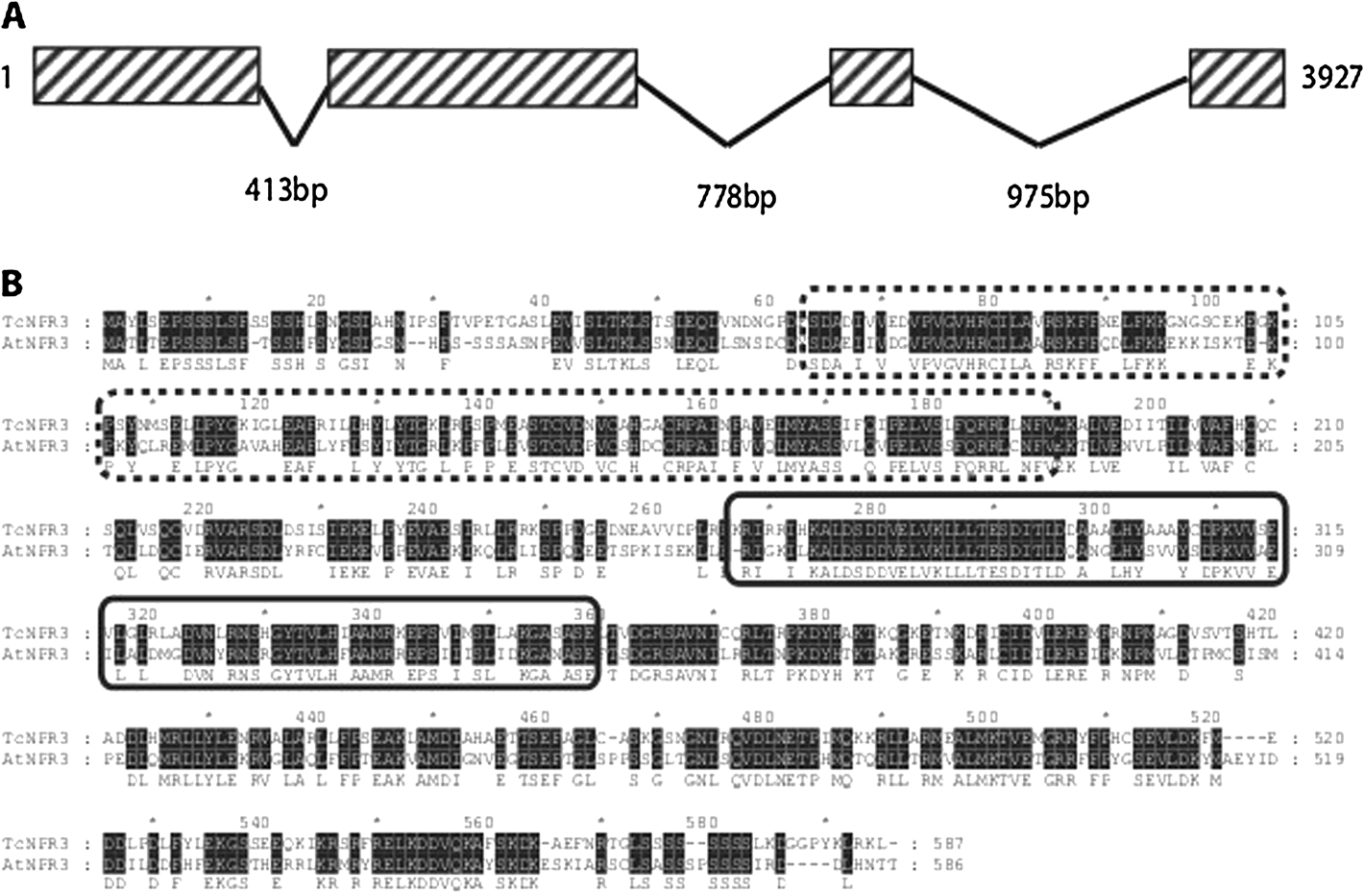
**Gene and protein structure of *Theobroma cacao *NPR3. A**. Diagram of the *TcNPR3* gene structure. Boxes with diagonal stripes represent exons, slanted lines represent introns. The sizes of the entire genomic fragment and each intron are indicated. **B**. Alignment of the Arabidopsis NPR3 and predicted *TcNPR3* proteins. Amino acids blocked in black indicate identical residues in both sequences and the amino acids below the blocks represent the consensus sequence. BTB/POZ and ankyrin repeat domains are highlighted by dashed lines and solid line boxes, respectively.

Subsequently, a genomic sequence containing a putative *TcNPR3* gene was identified by searching a cacao genome database (http://cocoagendb.cirad.fr/) [36] using the full-length cacao NPR3 cDNA as a query for the Blastn algorithm [38]. The structure of the *TcNPR3* gene (Tc06t011480) was deduced by comparing the full-length cDNA and genomic sequences using SPIDEY software tool (http://www.ncbi.nlm.nih.gov/spidey/index.html) [49], which revealed the presence of four exons and three introns, similar to the genomic structure of Arabidopsis *NPR3* (Figure 1A).

### Arabidopsis and cacao NPR3 protein sequences are highly similar

Conceptual translation of the *TcNPR3* predicted transcript resulted in a putative protein sequence consisting of 587 amino acid residues, only one amino acid longer than Arabidopsis NPR3. Alignment of the *TcNPR3* and *AtNPR3* protein sequences demonstrated that they are highly similar to each other (60% identity and 77% similarity). Arabidopsis and cacao NPR3 share key structural features (Figure 1B). Both proteins have a BTB/POZ domain near their N-terminus (dashed line box) that shares 64% identity and an ankyrin repeat region (solid line box) which shares about 72% identity. It has been shown that both BTB/POZ domain and ankyrin repeats are involved in protein-protein interactions [50-53]. These similarities in protein structure suggested that *TcNPR3* gene may also share the same function as *AtNPR3* in the regulation of the defense response.

### Expression of *TcNPR3* in cacao tissues

**Figure 2:**
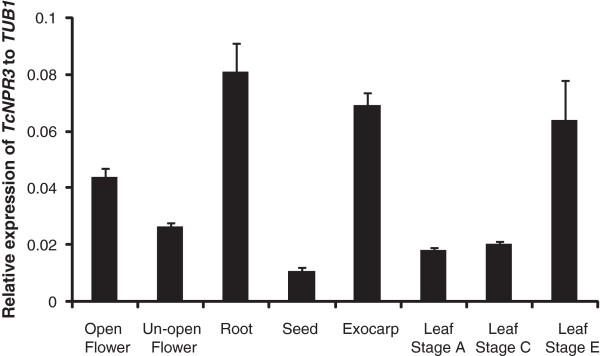
**Gene expression analysis of *TcNPR3 *in various cacao tissues.** Total RNA samples were collected from open flower, un-opened flower, root, seed, exocarp and three different leaf developmental stages from youngest to oldest (A, C and E) with three biological replicates for each tissue. RT-Q-PCR was performed using*TcTUB1*as a cDNA loading and normalization control. Expression levels are presented as the means ± standard errors of three biological replicates.

RT-Q-PCR was performed to investigate the expression level of *TcNPR3* in various cacao tissues, including leaves from sequential developmental stages A, C, E, representing young, mid and mature stages of development. In addition, RNA was isolated from open flowers, un-opened flowers, roots, seeds and fruit exocarp. *TcNPR3* is constitutively expressed at varying levels, in all tissues tested (Figure 2), similar to the Arabidopsis *NPR3* gene [23]. *TcNPR3* basal expression levels were relatively low in seed and moderate in floral tissues and young and mid-development leaves (stage A and C). Its expression was high in roots, exocarps and mature leaves (stage E).

### *TcNPR3* complements the Arabidopsis *npr3-3* mutation

Our earlier work has shown that the Arabidopsis *npr3* mutant phenotype includes a enhanced level of floral disease resistance and significantly reduced whole plant fitness [23]. To explore the function of *TcNPR3*, we introduced the cacao *NPR3* CDS under the control of a constitutive promoter into Arabidopsis *npr3-3* mutant transgenic plants, and tested its ability to complement the *npr3* mutant phenotype.

**Figure 3:**
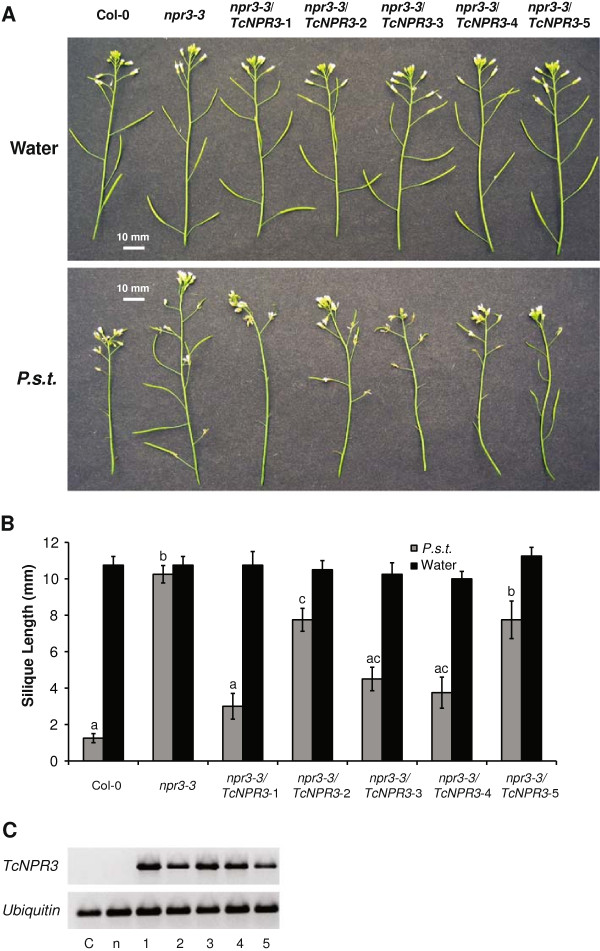
**Functional complementation of the Arabidopsis *npr3-3 *mutation by *TcNPR3*. ***Pseudomonas syringae* pv. tomato DC3000 infection assay of transgenic *npr3-3* mutant overexpressing *TcNPR3*. **A**. Representative images of siliques from infected Col-0, *npr3-3* mutant and five independent lines of *npr3-3* plants transformed with *TcNPR3* seven days after inoculation with virulent bacteria *Pseudomonas syringae* pv. tomato DC3000 (*P.s.t.*) (OD_600_ = 0.2 with 0.02% Silwet L-77) on top of the inflorescence. Additionally, water-treated (0.02% Silwet L-77) inflorescences of seven genotypes served as mock inoculation. Size bars are indicated in white (10 mm). **B**. Average lengths of the fourth silique from the bottom of infected inflorescences seven days after inoculation (gray bars) compared to water mock inoculations (black bars). Bar chart represents means ± SE of siliques from four biological replicates per treatment. Letters above the histogram indicate statistically significant differences among genotypes (P < 0.05) determined by single factor ANOVA. **C**. Gene expression of *TcNPR3* in transgenic Arabidopsis *npr3-3* lines. RT-PCR was performed with cDNA prepared from flowers of 6-week-old plants of wild type **(C)**, *npr3-3* mutant (n) and five individual transgenic *npr3-3* mutant overexpressing *TcNPR3* (1–5). Arabidopsis ubiquitin (*AtUbiquitin*) was assayed as an internal and cDNA loading control.

As compared to WT Arabidopsis, which is highly susceptible to *Pseudomonas syringae* pv. tomato DC3000 (*P.s.t.*), floral infection resulting in shortened siliques and reduced seed production, the primary phenotype of Arabidopsis *npr3-3* mutant is normal silique and seed development regardless of the inoculation of pathogen [23]. To test if *TcNPR3* overexpression in the *npr3-3* mutant can complement this phenotype, the floral infection assay was carried out with five individual *TcNPR3* transgenic lines, Col-0 and *npr3-3* mutant Arabidopsis plants. We inoculated young developing flowers with *P.s.t* and disease symptoms and bacterial growth were assayed. In water-treated inflorescences, we did not observe any phenotypic differences in any of the different lines (Figure 3A). Seven days after inoculation, the *P.s.t.*–treated Col-0 plants were more susceptible than *npr3-3* mutant plants, which showed impaired flower development and shorter siliques consistent with our previously reported results [23]. To evaluate the expression of the transgene, RT-PCR was performed using flowers of five independent transgenic lines along with wild-type Arabidopsis Col-0 and the *npr3-3* mutant controls. As expected, *TcNPR3* expression was not detected in either Col-0 or the *npr3-3* mutant, but five independent transgenic lines all showed heterologous expression of *TcNPR3* (Figure 3C). All five individual transgenic lines exhibited intermediate levels of silique development after pathogen inoculation relative to Col-0 and *npr3-3*. To quantify these differences, the length of siliques of each infected inflorescence was measured in four biological replicates (Figure 3B). The mean silique length of the *npr3-3* mutant was 11 mm regardless of the presence or absence of pathogen, but the silique length of infected Col-0 was significantly reduced to 1 mm. All five independent transgenic *npr3-3* lines overexpressing *TcNPR3* exhibited intermediate silique lengths, ranging from 3 mm to about 8 mm. The inoculated silique length of line 1 is statistically the same as Col-0, but all the other lines are significantly longer than Col-0 but shorter than in *npr3-3* mutant plants.

**Figure 4:**
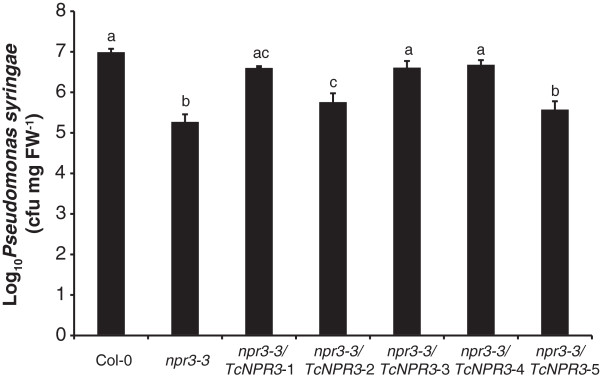
**Functional complementation of the Arabidopsis *npr3-3 *mutation by *TcNPR3*.** Bacterial populations of *P.s.t.* from infected flowers of Col-0, *npr3-3* mutant and five individual transgenic lines. The whole inflorescences of six-week old plants (genotypes as indicated on x-axis) were inoculated with virulent *P.s.t.* DC3000 (OD_600_ = 0.2 with 0.02% Silwet L-77) and bacterial growth was determined by bacterial titers five days after inoculation. Data represent means ± SE of four replicates, each containing two infected inflorescences from two individual plants. Letters above the histogram indicate the statistical differences among different genotypes (P < 0.05) determined by Fisher’s PLSD analysis. cfu, colony forming units.

To further evaluate the effect of the *TcNPR3* transgene in the complementation lines, we quantified bacterial titers in infected floral extracts. A decrease of bacterial colonization of about 60-fold was observed in the *npr3-3* mutant as compared to Col-0 (Figure 4). Although line 5 exhibited the same statistical level as *npr3-3* mutant, two of the transgenic lines (Line 3 and 4) showed a level close to that of Col-0. The other two lines (line 1 and line 2) also supported significantly more bacteria than *npr3-3* mutant though the level was not as high as in Col-0. In all, four out of five transgenic lines exhibited significantly higher bacteria levels than in *npr3-3* mutant, again suggesting that *TcNPR3* can at least partially complement *npr3-3* mutant phenotype. The *TcNPR3* protein only partially complemented the *npr3* mutant, most likely because of the heterologous nature of the interactions between *TcNPR3* and the Arabidopsis defense response machinery. It is also possible that *TcNPR3* has functions that differ from those of *AtNPR3*. To gain further evidence to support these conclusions, we tested *TcNPR3* function directly in *T. cacao*, using a transient expression system.

### Knockdown of *TcNPR3* in cacao leaves results in enhanced disease resistance

**Figure 5:**
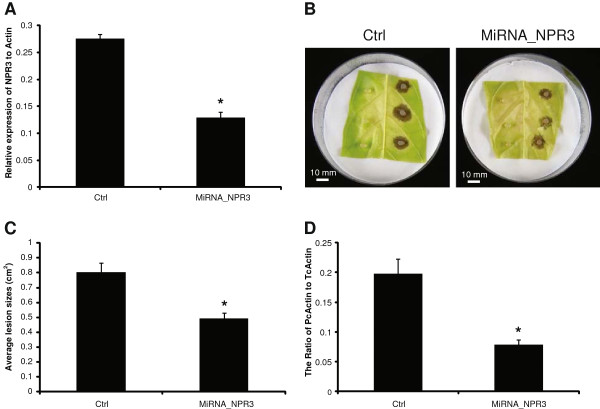
**Pathogen bioassays of cacao leaves transiently transformed with a *NPR3 *microRNA. A**. Relative expression of *NPR3* in cacao leaves by RT-Q-PCR. RNA samples were collected two days after transformation by *Agrobacterium* vacuum infiltration. Genotypes are indicated on x-axis as: Ctrl: control vector. MiRNA-NPR3: micro RNA of cacao NPR3 derived from mir319a in Arabidopsis. **B**. Representative images of transformed leaves three days after inoculation with agar plugs containing *Phytophthora capsici* mycelium on the right and agar plugs with water on the left. Size bars are indicated in white (10 mm). **C**. Average lesion sizes (mean ± SE of 12 lesion spots from four leaf discs). **D**. Relative virulence of *P. capsici* was measured by DNA RT-Q-PCR to calculate the ratio of pathogen actin to cacao actin three days after inoculation. Bars represent mean ± SE of four biological replicates each with three technical replicates. Asterisks show statistically significant difference to Ctrl determined by ANOVA.

Our expression data revealed that *TcNPR3* is expressed at moderate to high levels in leaves depending on developmental stage (Figure 2). To test the function of *TcNPR3* in cacao leaves, we knocked down its expression in cacao leaves by expressing an artificial microRNA via Agro-infiltration. We predicted that if NPR3 functions as a repressor of the NPR1-dependent defense response in cacao leaves, reduced *NPR3* transcript would result in increased pathogen resistance proportional to the NPR3 expression level. To test this hypothesis, a 21nt inverted repeat-hairpin construct was created based the design of Arabidopsis Mir319 [45] which was placed under control of an enhanced CaMV35S promoter. The artificial *TcNPR3* microRNA construct was expressed in cacao leaves by *Agrobacterium*-mediated transient transformation via vacuum infiltration. Native *NPR3* transcript levels were reduced up to 50% two days after transformation (Figure 5A). To explore the effect of *NPR3* expression on the ability of cacao leaves to respond and defend against pathogen infection, we inoculated the leaves with the cacao pathogen *Phytophthora capsici*. Three days after infection, lesions were formed in both control and NPR3-microRNA expressing leaf pieces (Figure 5B). Average lesion sizes were significantly reduced to about one-half those on controls (Figure 5C). To test the ability of pathogen to replicate on the leaves, we quantified the ratio of *Phytophthora* to cacao genomic DNA in the infection zones by RT-Q-PCR or specific target genes, which further demonstrated that the *TcNPR3*-mircoRNA transgene significantly reduced pathogenicity (Figure 5D).

## Discussion

The resources resulting from the complete genome sequence of *Theobroma cacao* were used to efficiently characterize the NPR1-like gene family of cacao [36]. We discovered a total of four *NPR-like* genes in the cacao genome. We previously reported that Tc09t007660 encodes TcNPR1, a functional ortholog of Arabidopsis NPR1 [35]. This report extends those findings to provide several lines of evidence supporting our hypothesis that Tc06t011480 (*TcNPR3)* encodes the functional ortholog of the Arabidopsis NPR3 gene. Two independent experiments testing *TcNPR3* function were consistent with a role in suppression of the NPR1-dependent defense response pathway (Figures 3, 4 and 5). The *TcNPR3* gene complemented the *npr3* mutant phenotype, which includes a dramatic increase in disease resistance. The fact that the complementation was only partial is not surprising, considering the large evolutionary distance of cacao and Arabidopsis. It is likely that specific protein-protein interactions between *TcNPR3* and Arabidopsis components such as NPR1, or TGA2 might not be as strong as in homologous interactions, and thus we observed only partial complementation. However, our data suggest that the mechanisms and molecules involved in NPR3-dependent signaling pathway in Arabidopsis are at least partially conserved with cacao. In a homologous test system, a cacao miRNA efficiently reduced endogenous *TcNPR3* mRNA expression, and this in turn resulted in increased resistance to pathogen infection, consistent with the function of NPR3 as a repressor of the defense response.

The pathogen used in our experimental bioassays, *P. capsici,* is considered to be a hemibiotrophic pathogen, and causes significant losses worldwide [54-56]. Our unpublished data showed over-expression of cacao NPR1 gene can increase the resistance to *P. capsici* in detached leaf assays, which is consistent with previous findings that NPR1-dependent pathway is mainly involved in defense against (hemi) biotrophic pathogens.

In Arabidopsis, the *npr3* knockout mutant exhibits a high level of pathogen resistance in developing flowers compared to Col-0, WT plantlets [23]. This level of resistance is correlated with reduced seed number and weight, likely the result of a physiological cost of heightened defense activation. This clearly illustrates how the cost of resistance drives the selective pressure for evolution of complex and efficient negative regulatory mechanisms. We hypothesize that NPR3 plays a similar role in cacao. Cacao *NPR3* gene expression is low in seeds, moderate in both floral tissues and young leaves, but elevated in the exocarps and stage E leaves (Figure 2). In contrast to the expression pattern or AtNPR3 in Arabidopsis, TcNPR3 only expressed at a moderate level in developing flowers, possibly because cacao is self-incompatible plant, so the tissue specificity might be different in this tropical tree. We speculate that evolution has selected for a mechanism that prioritizes resource allocation away from the mature exocarps and leaves, in favor of seeds, flowers and young leaves. This may play an important role for the pathogen *Moniliopthora perniciosa*, (witches’ broom disease) which can infect developing flower cushions and cause hypertrophy and abnormal fruit development.

These findings could be used to guide the screening of cacao germplasm for novel sources of disease resistance that could be incorporated into breeding programs. Screening of cacao varieties for lines expressing low levels of NPR3 transcript could be a rapid method for discovery of potentially resistant varieties. By defining the specific genes and mechanisms involved in cacao disease resistance, and translating these discoveries into tools for germplasm screening and breeding, it will be possible to pyramid multiple genes for resistance into the elite cacao genotypes of the future.

## Conclusion

The isolation of NPR3 from cacao and its heterologous complementation in Arabidopsis *npr3* mutant allowed us to rapidly confirm its function in the negative regulation of defense response in floral tissues. Moreover, the knock down of *TcNPR3* in cacao leaves via cacao transient transformation resulted in decreased resistance to cacao pathogen *Phytophthora capsici* in detached leaf bioassays. Taken together, we conclude that TcNPR3 is a functional ortholog of Arabidopsis NPR3 gene. This fundamental finding can potentially help us to breed cacao varieties with enhanced disease resistance by traditional breeding and/or transgenic manipulations, which will benefit farmers, developing countries, the environment and the chocolate industry.

## Competing interests

No financial or non-financial competing interests exist related to the subject matter of this manuscript. No reimbursements, fees, funding, or salary were received from any organization that may in any way gain or lose financially from the publication of this manuscript, at any time. All costs of this research and its publication charges were funded by grants from the Penn State Endowment in the Molecular Biology of Cacao. None of the authors hold stocks or shares in an organization that may in any way gain or lose financially from the publication of this manuscript, at any time. The authors are not currently applying for any patents relating to the content of the manuscript, nor have they received reimbursements, fees, funding, or salary from any organization that holds or has applied for patents relating to the content of the manuscript, There exist no non-financial competing interests (political, personal, religious, ideological, academic, intellectual, commercial or any other) to declare in relation to this manuscript.

## Authors’ contributions

ZS, SM and MG contributed to the design of all experiments, ZS was involved in implementation of most experimental plans and data acquisition, YZ contributed to the RT-Q-PCR experiment and data analysis of TcNPR3 expression across tissues, ZS and SM contributed to analysis and interpretation of all data as well as manuscript drafting and revision. MG conceived the overall plan of study, and participated in its design and coordination and helped to draft the manuscript. All authors read and approved the final manuscript.
